# Clinical validation of a computerized algorithm to determine mean systemic filling pressure

**DOI:** 10.1007/s10877-020-00636-2

**Published:** 2021-03-31

**Authors:** Loek P.B. Meijs, Joris van Houte, Bente C. M. Conjaerts, Alexander J. G. H. Bindels, Arthur Bouwman, Saskia Houterman, Jan Bakker

**Affiliations:** 1 Department of Intensive Care, Catharina Hospital, Eindhoven, The Netherlands; 2 Department of Cardiology, Catharina Hospital, Eindhoven, The Netherlands; 3 Department of Anesthesiology, Catharina Hospital, Eindhoven, The Netherlands; 4 Department of Anesthesiology, Maastricht University Medical Center, Maastricht, The Netherlands; 5 Department of Research and Education, Catharina Hospital, Eindhoven, The Netherlands; 6 Department of Intensive Care, Erasmus MC University Medical Centre, Rotterdam, The Netherlands; 7 Division of Pulmonary, Allergy, and Critical Care Medicine, Columbia University Medical Center, New York, NY USA; 8 Department of Pulmonary and Critical Care, New York University, New York, NY USA; 9 Department of Intensive Care, Pontificia Universidad Católica de Chile, Santiago, Chile

**Keywords:** Mean systemic filling pressure, Cardiac output, Right atrial pressure, Venous return, Inspiratory hold

## Abstract

Mean systemic filling pressure (Pms) is a promising parameter in determining intravascular fluid status. Pms derived from venous return curves during inspiratory holds with incremental airway pressures (Pms-Insp) estimates Pms reliably but is labor-intensive. A computerized algorithm to calculate Pms (Pmsa) at the bedside has been proposed. In previous studies Pmsa and Pms-Insp correlated well but with considerable bias. This observational study was performed to validate Pmsa with Pms-Insp in cardiac surgery patients. Cardiac output, right atrial pressure and mean arterial pressure were prospectively recorded to calculate Pmsa using a bedside monitor. Pms-Insp was calculated offline after performing inspiratory holds. Intraclass-correlation coefficient (ICC) and assessment of agreement were used to compare Pmsa with Pms-Insp. Bias, coefficient of variance (COV), precision and limits of agreement (LOA) were calculated. Proportional bias was assessed with linear regression. A high degree of inter-method reliability was found between Pmsa and Pms-Insp (ICC 0.89; 95%CI 0.72–0.96, *p* = 0.01) in 18 patients. Pmsa and Pms-Insp differed not significantly (11.9 mmHg, IQR 9.8–13.4 vs. 12.7 mmHg, IQR 10.5–14.4, *p* = 0.38). Bias was −0.502 ± 1.90 mmHg (*p* = 0.277). COV was 4% with LOA –4.22 − 3.22 mmHg without proportional bias. Conversion coefficient Pmsa ➔ Pms-Insp was 0.94. This assessment of agreement demonstrates that the measures Pms-Insp and the computerized Pmsa-algorithm are interchangeable (bias −0.502 ± 1.90 mmHg with conversion coefficient 0.94). The choice of Pmsa is straightforward, it is non-interventional and available continuously at the bedside in contrast to Pms-Insp which is interventional and calculated off-line. Further studies should be performed to determine the place of Pmsa in the circulatory management of critically ill patients. (www.clinicaltrials.gov; TRN NCT04202432, release date 16-12-2019; retrospectively registered).

*Clinical Trial Registration* www.ClinicalTrials.gov, TRN: NCT04202432, initial release date 16-12-2019 (retrospectively registered).

## Introduction

Mean systemic filling pressure (Pms) is a promising parameter in determining effective intravascular fluid status, which is key in the daily treatment of the critically ill [1–7]. The concept of Pms has been extensively investigated by Guyton et al., in which its role in venous return and volume responsiveness is integrated with the existing knowledge of cardiac contractile reserve from the Frank-Starling theorem. However, despite several decades of research, no easy clinical way of measuring Pms has been developed. To measure Pms the intravascular equilibrated no-flow pressure, as performed by Guyton et al. in the past, is necessary [1–3, 8–10]. This method of assessing Pms is not attainable in clinical patients. Pms derived from venous return curves created by performing inspiratory holds during incremental airway pressures (Pms-Insp) was found to be a clinically feasible method [11–16]. Parkin, based on animal studies by Guyton, proposed an algorithm to calculate an analogue of Pms (Pmsa) using the main parameters right atrial pressure (RAP), mean arterial pressure (MAP) and cardiac output (CO) [17–22]. Maas et al. found a good correlation between Pmsa and Pms-Insp, however this Pmsa was calculated offline after study procedures. Moreover, they found considerable bias between the two methods and the necessity for a conversion coefficient to match Pmsa values to Pms-Insp values. Since the Pms-Insp technique is laborious and cumbersome in clinical practice, a more practical alternative is required. Recently, the algorithm to calculate Pmsa has been made publicly available which enables physicians to generate real-time bedside Pms-values. This study was designed to compare realtime computerized Pmsa-values with Pms-Insp deducted from venous return curves during inspiratory hold maneuvers as the reference method in post-cardiac surgery patients.

## Methods

This prospective observational study was conducted at the Intensive Care Unit (ICU) of the Catharina Hospital, Eindhoven, The Netherlands. Prior to patient enrollment this study was approved by the Medical research Ethics Committees United, Nieuwegein, The Netherlands at March 6th 2019 (NL67389.100.18, R18.070) and was registered at ClinicalTrials.gov (NCT04202432). All patients signed informed consent before study inclusion. The study adhered to the principles of the Declaration of Helsinki (64th WMA General Assembly, Fortaleza, Brazil, October 2013) and was in accordance with the Medical Research Involving Human Subjects Act (WMO). The guidelines for Standards for Reporting Diagnostic Accuracy (STARD) were applied in preparation of this article [23].

### Patients

Patients who underwent elective coronary artery bypass grafting (CABG) or off-pump coronary artery bypass grafting (OPCAB) were eligible for this study. Patients had to be 18 years or older with a left and right ventricular ejection fraction (LVEF/RVEF) of 50% or more and no valvular insufficiency or stenosis. Patients with severe comorbidities were not eligible for this study. There had to be no previous history of pulmonary disease (e.g. previous pneumonectomy, lobectomy, moderate to severe chronic obstructive pulmonary disease (COPD) stage 3–4 or acute respiratory distress syndrome (ARDS). Patients with severe peripheral artery disease, aortic aneurysm, postoperative arrhythmia, indication for external pacing or a cardiac assist device were excluded. If moderate to severe bleeding was present postoperatively (drain production >50 mL/15 min) patients were also excluded.

Pre-operatively a pulse-contour CO catheter (PiCCO^®^ v6.0, Getinge AB, Gothenburg, Sweden) was inserted in the femoral artery [24]. As part of standard of care, a central venous catheter was inserted in the right jugular vein. After surgery, patients were admitted to the ICU. Post-operatively sedation was maintained to perform successful measurements and minimize patient discomfort. Mechanical ventilation was kept on pressure-controlled ventilation (PCV) with a positive end-expiratory pressure (PEEP) of 5 cm H_2_O and tidal volumes between 6 and 8 mL/kg (Hamilton G5/S1, Hamilton Medical AG, Bonaduz, Switzerland). During study procedures all subjects were hemodynamically stable and no changes in vasoactive medication were made. No fluids were administered during the study protocol.

### Measurements

Patients were connected to the standard ICU monitor in our department (MP70, Philips^®^, Best, The Netherlands) for hemodynamic and respiratory observation. Arterial and venous pressure transducers were referenced to the mid-axillary line at the level of the right atrium. PiCCO^®^ was connected to the PiCCO^®^-2 monitoring module (Getinge AB, Gothenburg, Sweden). A set of three thermodilution derived measurements (20 mL saline, 7 °C) was performed to calibrate PiCCO^®^. Calibration was accepted if thermodilution-derived CO measurements did not show outliers with >10% difference in CO from the mean of triplet measurements. Once calibrated, continuous pulse-contour derived CO measurements were used for this study. RAP, MAP and CO data were continuously transferred from the Philips^®^- and PiCCO^®^-2-monitors to a bedside monitor with the embedded computerized Pmsa-algorithm in order to calculate mean systemic filling pressure in realtime (Navigator™ Clinical Decision Support System, supplied as NaviCorder by CPL Innovations Pty Ltd., Sydney, NSW, Australia). The methods of the Pmsa-algorithm have been described in detail before [17–19, 25]. In short, it uses the formula Pmsa = (0.96 · RAP) + (0.04 · MAP) + (c · CO), were c has elements of vascular resistance combined with patient’s age, height and weight (c = 0.038 · (94.17 + 0.193 · age)/(4.5 · [0.99^(age – 15^)] · 0.007184 · [height^0.725^] · [weight^0.425^])). All data were continuously logged by the PiCCO^®^ and Pmsa-monitors. Study interventions according to the protocol were consistently tagged on the monitors for manual read-out afterwards.

### Protocol

The Pmsa-value recorded before onset of inspiratory hold procedures was used for comparison with Pms-Insp. Consecutively, stepwise increases in airway plateau pressure (Pplateau) from 5 to 10–15-20-25-30 cm H_2_O with 2-min intervals by changing pressure control settings on the ventilator with respect to tidal volumes <1000 mL/breath, were applied. Each level of Pplateau was followed by an inspiratory hold of at least 12 s. This method has been previously described [11–13, 15, 16]. CO, cardiac index (CI) and RAP were recorded during these maneuvers and plotted offline to construct venous return curves. The intersect of the RAP at zero flow CI indicated mean systemic filling pressure.

### Statistical analysis

Data-analysis was performed based on the study objective described in the introduction and was defined before initiation of data acquisition. The Kolmogorov-Smirnov test was used to assess normality. Continuous (numeric) variables were summarized (depending on normality) by mean, median, standard deviation (SD), interquartile range (IQR; 25th and 75th percentiles) and range (minimum, maximum). Categorical variables were represented by absolute and relative frequencies.

Venous return curves were constructed by linear regression analysis using the least squares method to determine Pms-Insp. Differences in median values of Pmsa and Pms-Insp were assessed with the Wilcoxon signed rank test. Intraclass correlation coefficient (ICC) using the two-way mixed model was calculated to test absolute agreement between Pmsa and Pms-Insp. A Bland-Altman analysis was performed in order to test bias, precision and limits of agreement (LOA). Bias was defined as the mean difference between Pms-Insp and Pmsa. Precision was defined by the standard deviation (SD) of these differences. LOA were set at bias ±1.96 SD. Linear regression analysis of Bland-Altman was used for testing proportional bias. Coefficient of variance was calculated as 100% · (SD/mean). To correct for possible bias by vasopressor medication, absolute values of Pms between the groups with or without vasopressors were tested with the Mann-Whitney U test. Also, ICC and Bland-Altman-analyses were performed in these separate groups. A *p* value <0.05 was considered to be statistically significant. All analyses were performed using SPSS Statistics for Macintosh, version 26.0 (IBM Corporation, Armonk, New York, USA) and Graphpad Prism, version 8.2.1 (Graphpad Software Inc., San Diego, California, USA).

## Results

**Fig. 1:**
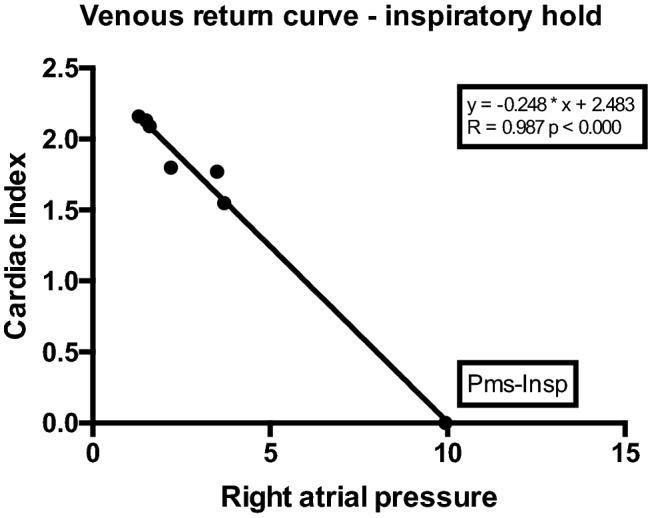
Example of venous return curve. Data points plotted represent consecutive cardiac index (CI) (y-axis) and corresponding right atrial pressure (RAP; x-axis) values during 12 s inspiratory hold maneuvers. With each increment of airway plateau pressure (Pplateau), CI (or venous return; VR; as in steady state conditions VR determines CI) will decrease, whereas RAP will increase. Pms-Insp (Pms calculated after inspiratory hold maneuver) is calculated by extrapolation of the VR-curve with linear regression (least squares method). The intersect of the VR-curve with the x-axis (at zero CI or VR) represents true Pms-Insp

**Fig. 2:**
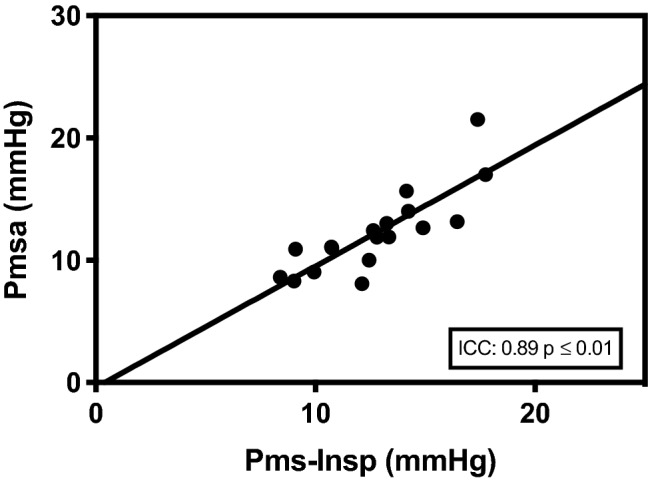
Intraclass correlation of Pmsa and Pms-Insp. Association between realtime Pms calculated by computerized algorithm (Pmsa) and Pms calculated after inspiratory hold maneuver (Pms-Insp). Intraclass correlation coefficient (ICC) is presented in the lower right corner of the scatter plot (95% CI 0.72–0.96, *p* ≤ 0.01)

**Fig. 3:**
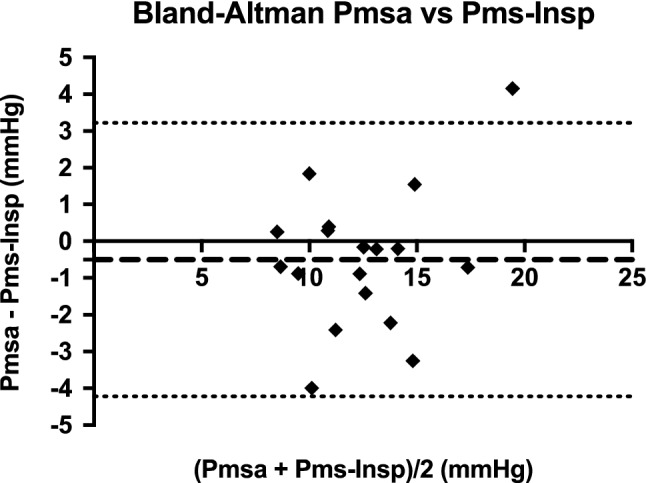
Bland-Altman analysis of Pmsa and Pms-Insp. Bland-Altman analysis showing the comparison between measurements of realtime Pms calculated by computerized algorithm (Pmsa; test-method) and Pms calculated from venous return curves during inspiratory hold maneuvers (Pms-Insp; reference method). The dashed horizontal line represents the mean of the differences (bias) which was found to be −0.502 ± 1.90 mmHg, *p* = 0.277. The upper and lower dotted horizontal lines represent the 95% limits of agreement (LOA) which are −4.22 and 3.22 mmHg

In total 18 patients were included in this study. Baseline characteristics of patients are presented in Table 1. Venous return curves were constructed using linear regression analysis in all patients. An example is shown in Fig. 1. There was no statistical difference between median Pmsa and median Pms-Insp (11.9 mmHg, IQR 9.8–13.4 vs. 12.7 mmHg, IQR 10.5–14.4 respectively, *p* = 0.07). A high degree of reliability was found between the Pmsa and Pms-Insp with an average measured ICC of 0.89 (95% CI 0.72–0.96, *p* = 0.01) (Fig. 2). Pmsa and Pms-Insp showed a bias of −0.502 ± 1.90 mmHg (*p* = 0.28), with a COV of 4% and LOA −4.22 and 3.22 mmHg (Fig. 3). Regression analysis of the Bland-Altman plot revealed no proportional bias between the two measurement methods (B coefficient = 0.201, *p* = 0.15). The mean coefficient to calculate Pms-Insp from Pmsa was 1.06 ± 0.16, implying that Pmsa is a factor 0.94 in comparison to Pms-Insp.

**Table 1:** Baseline characteristics

Patient characteristics	Data (*n* = 18)
Age (y)	63 (35–78)
Gender (M/F)	14/4
Height (cm)	176 (156–191)
Body weight (kg)	86 (61–110)
Body surface area (m^2^)	2.02 (1.61–2.24)
*Surgery*	
CABG/OPCAB	13/5 (72%/18%)
Perioperative fluid administration (mL)	4210 ± 2169
Perioperative Fluid Balance (mL)	2439 ± 1213
*Vasoactive and sedative drugs*	
Norepinephrine (*n*; dosage μg/kg/min)	(5/18); 0.02 (0–0.20)
Phenylephrine (*n*; dosage μg/kg/min)	(3/18); 0.03 (0–0.27)
Propofol (*n*; dosage mg/h)	18/18); 253 (150–400)
*Hemodynamic baseline data*	
Cardiac Index (mL/min/m^2^)	2.65 (1.90–4.30)
Right atrial pressure (mmHg)	4.40 (1.90–10.50)
Mean arterial pressure (mmHg)	69 (54–87)
*Ventilator settings*	
Tidal volume (mL/kg predicted body weight)	7.63 (4.15–10.66)
Respiratory rate (breath/min)	13 ± 2
Total PEEP (cm H_2_O)	5 ± 0
FiO_2_ (%)	40 ± 0

Values are expressed as mean ± standard deviation, median (25th–75th percentile) or absolute numbers with percentages or ranges, as appropriate. *CABG* coronary artery bypass grafting, *F* female, *FiO*_*2*_ inspired oxygen fraction, *M* male, *OPCAB* off-pump coronary artery bypass grafting, *PEEP* positive end-expiratory pressure

Eight out of 18 patients were treated with vasopressors, of which the dosage remained unchanged during study procedures. In this subset of patients, no significant difference between median Pmsa and median Pms-Insp could be detected (13.3 mmHg, IQR 11.2–16.7 vs. 14.2 mmHg, IQR 11.3–16.8 respectively, *p* = 0.84). Again, in these patients a high degree of reliability between Pmsa and Pms-Insp was found with an ICC of 0.92 (95% CI 0.59–0.98, *p* ≤ 0.01). Bias was found to be −0.110 ± 1.95 mmHg (*p* = 0.878) with a COV of 17% and LOA −3.93 and 3.71 mmHg. Absence of proportional bias was confirmed by regression analysis of the Bland-Altman plot (B coefficient = 0.355, *p* = 0.12).

In those patients without vasopressors (*n* = 10), median Pmsa and median Pms-Insp did not differ (11.0 mmHg, IQR 8.5–12.6 vs. 12.3 mmHg, IQR 9.3–13.3 respectively, *p* = 0.08). ICC showed a good reliability between Pmsa and Pms-Insp (ICC 0.76, 95% CI 0.11–0.94, *p* = 0.01). Bias was −0.992 ± 1.80 mmHg (p = 0.12) with a COV of 2% and LOA −4.52 and 2.54 mmHg. Finally, regression of the Bland-Altman plot also showed no proportional bias (B coefficient = 0.297, *p* = 0.35).

No significant difference between the median values of Pmsa in patients with or without vasopressors was present (13.3 mmHg, IQR 11.2–16.7 vs. 11.0 mmHg, IQR 8.5–12.6, p = 0.08). Neither was there a difference between the median values of Pms-Insp (14.2 mmHg, IQR 11.3–16.8 vs. 12.3 mmHg, IQR 9.3–13.3, *p* = 0.20).

## Discussion

This study shows a good correlation between Pmsa and Pms-Insp. There was no significant difference between the Pms-Insp and Pmsa values. Pmsa appeared to be a reliable reflection of Pms-Insp (ICC 0.89, *p* = 0.01) without bias. Given the fact that the COV is 3% in the total of 18 patients indicates that there is very limited variation between Pmsa and Pms-Insp.

The algorithm for calculating Pmsa has been tested in previous studies, however this was performed in an offline setting without using realtime data [13, 26]. They showed that the algorithm correlated well with Pms-Insp and moreover that the Pms-algorithm followed the same trend as Pms-Insp when patients were subjected to passive leg raising maneuvers or fluid challenges. Nevertheless, there was considerable bias between the two methods and therefore a conversion coefficient was introduced to convert Pmsa to Pms-Insp. Despite the fact that Pms proved to be a promising index of volume state (and volume responsiveness), the laborious technique of Pms-Insp hampers clinical use of Pms the circulatory management of patients.

Our study shows that with standard invasive techniques (indwelling CO and central venous catheters), an adequate estimation of Pms can be made. Therefore, the Pmsa can be used at the bedside for measuring Pms in both patient care and study purposes. For this study we used the Navigator™-monitor with the embedded Pmsa-algorithm to calculate Pmsa in realtime. This monitor is not commercially available anymore. However, since the algorithm to calculate Pmsa has become publicly available, it could be applied in any bedside monitor that measures CI, MAP and RAP.

Now that Pmsa is able to reliably measure mean systemic filling pressure, volume status may be adequately predicted in critically ill patients. Maas et al. showed that following passive leg raising maneuvers and fluid challenge Pms-Insp and Pms-algorithm increased in a similar trend. In their studies a coefficient of 0.7 was found to convert Pms-algorithm to Pms-Insp [13]. This conversion coefficient was 0.94 in our study, showing that Pmsa is nearly equal to Pms-Insp.

Performing a study in postsurgical patients using Pmsa, Cecconi et al. found that changes in Pms were well correlated with changes in CO and the pressure gradient for venous return (pVR; the driving pressure for preload). However, they did not validate Pmsa in comparison to the reference method [27]. Rangappa et al. found that Navigator™ as a clinical decision-support system improved consistency between clinicians in treating patients, however validation of Pmsa was not part of this study protocol [28].

Guyton et al. concluded that a normal Pms was defined between 7 and 12 mmHg in repeated animal studies [1–3, 8]. We found similar values in our population (Pmsa 11.9 mmHg vs. Pms-Insp 12.7 mmHg). This was lower than the mean Pms observed in the studies by Maas et al. [13, 15]. A recent study by Repessé et al. measuring Pms in deceased patients 1 min after the heart stopped beating, the equilibrium of arterial and venous pressures was 12.8 ± 5.6 mmHg, which is in line with Pmsa and Pms-Insp values in our study [29]. However, these values seem to be at the upper limit of normal as concluded by the animal experiments by Guyton. In our population the mean fluid infusion from induction of anesthesia until the end of surgery was 4210 mL with a mean fluid balance of +2438 mL. This might explain the relatively high Pms-values. On the other hand, all patients were sedated with propofol which is known to lower the Pms [30].

Measurements of Pms in a porcine model conducted by Berger and colleagues showed that venous return curves deducted from inspiratory hold procedures with moderate levels of PEEP overestimated Pms [31]. Our study differs from this protocol since incremental airway plateau pressures were modified in contrast to PEEP levels, showing slightly lower Pms values which are in line with current and seminal literature [1–4, 8, 10, 11, 29]. Another study focused on the effect of PEEP and tidal ventilation on Pms in deceased patients showed a small but significant increase in Pms as well [32]. Despite indications that PEEP and tidal volumes influence Pms, both Pmsa as Pms-Insp were measured under the same PEEP-levels and tidal volumes during our protocol. Both parameters correlated well without significant bias.

Maas and Persichini showed that norepinephrine affects CO and MAP [33, 34]. So, in theory, norepinephrine might influence Pmsa since CO and MAP are part of the algorithm that is used. However, in our study we did not find significant differences in the estimated values of Pmsa with or without vasopressors. Neither was there a difference between these groups in Pmsa versus Pms-Insp.

### Limitations

Determining Pms from venous return curves via inspiratory breath holds has its flaws. First of all, true Pms cannot be measured by this means. As mentioned before, hereto a stop-flow situation should be created which is not feasible nor ethical in humans. To the best of our knowledge, Pms-Insp was chosen to serve as an interchangeable method to compare Pmsa with. Pms-Insp has been tested in canine studies under controlled circumstances, showing a good correlation with zero-flow Pms [11]. The analogue method by Parkin and Leaning was also studied in a dog model of endotoxemia using pulmonary artery flow probes and pleural pressure measurements. In this retrospective analysis from previous study results, the investigators compared the analogue to the instantaneous venous return curve method and concluded that changes in the analogue estimate of Pms accurately tracked the Pms as estimated from the instantaneous venous return curves [35, 36]. More recent studies by Berger and Moller reported that Pms-Insp either overestimated or underestimated Pms determined by right atrial balloon occlusion (as a surrogate for stop-flow Pms) respectively [31, 37]. From the literature it seems that Pmsa at least gives an estimate of Pms and that it tracks changes in volume status accurately.

With ventilatory adjustments during the inspiratory hold procedures, there is a chance of exciting the cardiopulmonary or autonomic nervous system. We did not observe significant changes in heart rate during all study procedures. Cardiac output, RAP and MAP all returned to baseline values once Pplateau was put back to initial settings. Moreover, during the studies with instantaneous VR-curves by Pinsky et al., a 1-min infusion followed by a 5-min stabilization period was used. This was considerably longer than our 2-min interval, however no new equilibrium was observed during these experiments [11, 35, 36].

In our study CO was measured at the left side of the heart. Technically, these measurements may differ from CO-values measured at the right side of the heart, due to heart-lung interactions during positive pressure ventilation [38, 39] This could have influenced our Pms-Insp values.

Although this study shows that Pmsa is a reliable reflection of Pms-Insp without bias, this protocol did not focus on determining whether both indices follow the same trend when performing repeated measurements of Pms. In the study by Maas et al. the dynamic changes in Pmsa and Pms-Insp have been shown to correlate well. Our study was performed in stable post-cardiac surgery patients. For clinical applicability, Pmsa should also be validated in other patient categories. Furthermore, the small sample size could be a limiting factor for interpreting the results of this study. For this purpose, we have performed a post-hoc power analysis using the intraclass correlation coefficient (ICC) of this study. Using an ICC of 0.89 as obtained in this study, a null hypothesis value for the ICC of 0, two ratings for each subject, an alpha of 0.05 and a 2-tailed test, an estimated power of 0.99 was found.

However, although the bias in our Bland-Altman analysis is −0.502 mmHg, the LOAs are −4.22 to 3.22 mmHg, which, from a clinical perspective, is rather wide given the reference values of Pms are 7–12 mmHg as found by Guyton et al. [1–3, 8]. This implies that our findings are accurate but could be imprecise, possibly due to the small population used in this study.

This study has a prospective, observational design. No outcome related measures can be concluded from our findings. In order to do so, future double-blinded randomized controlled studies should be performed.

## Conclusion

Mean systemic filling pressure calculated by the Pmsa-algorithm is a reliable method for determining Pms in post-cardiac surgery patients at the bedside. Further studies should be performed to determine the place of Pmsa in the circulatory management of critically ill patients.

## Data Availability

Reviewers can be provided with data upon request.
